# A simplified procedure to trace triglyceride‐rich lipoprotein metabolism *in vivo*

**DOI:** 10.14814/phy2.14820

**Published:** 2021-05-04

**Authors:** Zhixiong Ying, Mariëtte R. Boon, Tamer Coskun, Sander Kooijman, Patrick C. N. Rensen

**Affiliations:** ^1^ Department of Medicine Division of Endocrinology Einthoven Laboratory for Experimental Vascular Medicine Leiden University Medical Center Leiden the Netherlands; ^2^ Department of Diabetes/Endocrine Lilly Research Laboratories Lilly Corporate Center Indianapolis IN USA

**Keywords:** lipoprotein kinetics, lipoprotein metabolism, lipoproteins, plasma triglyceride metabolism, VLDL

## Abstract

Glycerol tri[^3^H]oleate and [^14^C]cholesteryl oleate double‐labeled triglyceride‐rich lipoprotein (TRL)‐like particles are a well‐established tool to trace the effect of lipid‐modulating interventions on TRL metabolism. The routine generation of these particles involves sonication of a lipid mixture and subsequent fractionation of resulting particles into populations of different average size through density gradient ultracentrifugation. Here, we describe a simplified and more time‐efficient procedure for preparing TRL‐like particles without the need of fractionation. The simplified procedure shortened the preparation of particles from over 4 h to less than 2 h and generated particles with a higher yield, although with a smaller average size and more heterogeneous size distribution. In C57Bl/6J mice housed at thermoneutrality (30°C), the two preparations showed highly comparable plasma clearance and organ distribution of glycerol tri[^3^H]oleate‐derived [^3^H]oleate and [^14^C]cholesteryl oleate, as measures of lipolysis and core remnant uptake, respectively. Upon a cold challenge (14°C), plasma clearance was accelerated due to enhanced uptake of glycerol tri[^3^H]oleate‐derived [^3^H]oleate by brown adipose tissue. The simplified procedure resulted in a modestly increased particle uptake by the spleen, while uptake by other organs was comparable between the two preparations. In conclusion, the simplified procedure accelerates the preparation of TRL‐like particles for tracing *in vivo* TRL metabolism. We anticipate that this time‐efficient procedure will be useful for incorporation of PET‐traceable lipids to obtain more insight into human lipoprotein metabolism.

**New & Noteworthy**

We present a simplified and time‐efficient procedure to prepare triglyceride (TG)‐rich lipoprotein‐like particles to track TG‐rich lipoprotein metabolism *in vivo*. This adjusted procedure raises the possibility of more broad application of this technique and incorporation of short‐lived PET‐traceable lipids to non‐invasively obtain more insight into human lipoprotein metabolism.

## 1 INTRODUCTION

Chylomicrons and VLDL are the triglyceride (TG)‐rich lipoproteins (TRLs) that are produced by the intestine and liver, respectively, in order to deliver TG‐derived fatty acids (FAs) to peripheral tissues for storage (white adipose tissue; WAT) or combustion to generate heat (brown adipose tissue; BAT) and ATP (e.g., skeletal muscle, heart). In the circulation, TGs are hydrolyzed by lipoprotein lipase (LPL) bound to endothelial cells of capillaries in these metabolically active tissues (Goldberg, 1996). The vast majority of the released FAs are taken up by underlying parenchymal cells (i.e., adipocytes, myocytes) via the cluster of differentiation 36 (Goldberg et al., 2009) and FA transport proteins (Doege & Stahl, 2006); the remainder associates in plasma with albumin and is shuttled to the liver (Teusink et al., 2003). During lipolysis, TRLs get enriched with apolipoprotein E, through which the TRL remnants are recognized by the LDL receptor or LDL receptor‐related protein for uptake, primarily by the liver (Miller, 1979).

In 1995, Rensen et al. (1995) reported on a method to generate approx. 75‐nm‐sized TRL‐like particles for selective delivery of amphiphilic drugs to the liver. Incorporation of a derivate of the nucleoside analog iododeoxyuridine, an antiviral drug used in the treatment of hepatitis B infection, into these particles led to a 40‐fold increased liver uptake in addition to markedly reduced extrahepatic deposition as compared to administration of the free drug (Rensen et al., 1995). In subsequent years, we have exploited these TRL‐like particles mainly to study the mechanisms underlying lipid‐modulating strategies. For example, we have used glycerol tri[^3^H]oleate ([^3^H]triolein; [^3^H]TO) and [^14^C]cholesteryl oleate ([^14^C]CO) incorporated in TRL‐like particles to show that pharmacological/physiological activation of BAT leads to increased LPL‐mediated [^3^H]TO‐derived oleate uptake by BAT coupled to enhanced clearance of the [^14^C]CO‐labeled core remnants by the liver (Berbee et al., 2015; Khedoe et al., 2015), resulting in an anti‐atherogenic lipoprotein profile (Berbee et al., 2015).

The procedure employed to generate TRL‐like particles requires density gradient ultracentrifugation and is therefore rather time consuming. Here, we describe a simplified and more time‐efficient procedure for the preparation of TRL‐like particles, anticipating the future use of these particles for non‐invasive imaging of TRL metabolism using short‐lived PET tracers.

## 2 MATERIALS AND METHODS

### 2.1 Chemicals

Glycerol trioleate (triolein; TO; ≥99%), L‐α‐lysophosphatidylcholine (≥99%), cholesteryl oleate (CO; ≥98%), and cholesterol (≥99%) were purchased from Sigma‐Aldrich. Egg yolk phosphatidylcholine (98%) was obtained from Lipoid (Switzerland). [^3^H]TO and [^14^C]CO were obtained from PerkinElmer.

### 2.2 Preparation of radiolabeled TRL‐like particles using the routine procedure

The routine procedure was previously described by Rensen et al. (1997). Briefly, TRL‐like particles were prepared from 100 mg of total lipid including TO (70 mg), egg yolk phosphatidylcholine (22.7 mg), lysophosphatidylcholine (2.3 mg), CO (3.0 mg), and cholesterol (2.0 mg), with the addition of [^3^H]TO (100 μCi) and [^14^C]CO (10 μCi). After drying the lipids (dissolved in 1:1 v/v CH_3_OH:CHCl_3_) in a 20‐ml glass flat‐bottom vial by a gentle stream of N_2_, the lipid mixture was sonicated in 10 ml of NaCl buffer (density: 1.10 g/ml) containing 10 mM HEPES and 1 mM EDTA, pH 7.4, for 2 × 15 min, using a Soniprep 150 (MSE Scientific Instruments) equipped with a water bath at 54°C, at 10 μm output. Then, the crude emulsion (10 ml) was divided over two ultracentrifugation tubes (5 ml each), overlayed with HEPES/EDTA NaCl buffers with sequential densities of 1.06 g/ml (2.6 ml), 1.02 (2.6 ml), and 1.006 g/ml (approx. 2.6 ml), and fractionated by ultracentrifugation (SW 40 Ti swinging bucket rotor). After the first run of 27 min at 20,000 rpm (approx. 71,000 g) at 20°C, the top lipid layer containing large TRL‐like particles (with an average diameter around 150 nm) was discarded. After the second run of 27 min at 40,000 rpm (approx. 285,000 g) at 20°C, a second fraction containing particles with an average diameter around 75 nm was collected. This particle emulsion was stored at 4°C under argon and used for experiments within 5 days after preparation.

### 2.3 Preparation of radiolabeled TRL‐like particles using a simplified procedure

After combining and drying lipids as described above, the crude emulsion was sonicated in 10 ml regular saline followed by brief centrifugation (1 min; 20 g; 20°C) to remove titanium fragments derived from the sonotrode tip, stored at 4°C under argon, and used within 5 days after preparation.

### 2.4 Measurement of the size of TRL‐like particles

The average size, size distribution, and homogeneity of the TRL‐like particles that were prepared by both procedures were measured by dynamic light‐scattering using a fixed‐angle Zetasizer Nano ZSP (Malvern Instruments).

### 2.5 Animal experiment

The mouse experiment was performed in accordance with the Institute for Laboratory Animal Research Guide for the Care and Use of Laboratory Animals and has received approval from the Central Animal Experiments Committee of the Netherlands. To resemble the human situation, we performed experiments under thermoneutral conditions, namely 30°C (equivalent to 21°C for humans) and performed a cold stress test at 14°C (equivalent to 5°C for humans). Eight‐week‐old male C57Bl/6J mice (Charles River Laboratories, St. Germain Nuelles) were housed in a 12: 12 h light‐dark cycle at thermoneutrality (30°C) with free access to chow and water. After 3 weeks of acclimatization, the mice were single housed and exposed to a daily 4‐h cold challenge (14°C) or kept at thermoneutrality (30°C) while being fasted. After 1 week, 4 h into the cold challenge and/or fasting, half of each group was intravenously injected via the tail vein with 200 µl (i.e., equivalent to 1 mg TG) of the emulsion containing either routinely prepared TRL‐like particles or TRL‐like particles prepared according to the simplified procedure.

### 2.6 *In vivo* plasma decay and organ uptake of TRL‐like particles

Blood samples were taken from the tail vein at 2, 5, 10, and 15 min after TRL‐like particles were injected to determine the plasma decay of [^3^H]TO and [^14^C]CO. Plasma volumes were calculated as 0.04706 × body weight (g) (Jong et al., 2001). Then, mice were euthanized by CO_2_ inhalation and perfused with ice‐cold PBS to remove the blood and noninternalized particles from organs. Finally, organs and tissues were collected, (part of) organs and tissues (approx. 70 mg) were weighed and dissolved overnight at 55°C in Solvable (Perkin Elmer), and ^3^H and ^14^C activity were quantified in Ultima Gold liquid scintillation cocktail. Uptake of [^3^H]TO‐ and [^14^C]CO‐derived radioactivity by the organs was expressed per gram wet tissue weight.

### 2.7 Statistics

All data are presented as mean ± SEM and analyzed by unpaired Student's *T*‐test with GraphPad Prism 8. *p < 0.05 was considered statistically significant.

## 3 RESULTS

### 3.1 Yield and size distribution of TRL‐like particles prepared using the simplified versus routine preparation procedure

**FIGURE 1:**
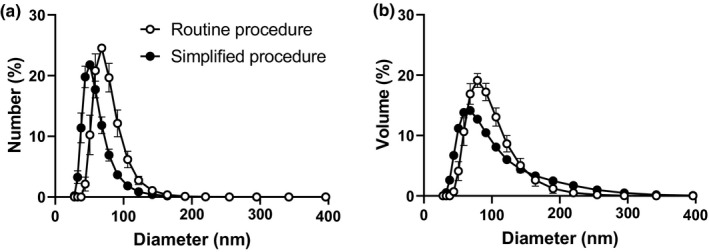
Simplified procedure generates TRL‐like particles with smaller size. Distribution of particle number (a) and volume (b) of independent TRL‐like particle preparations was measured using the Zetasizer Nano ZSP. Values are means ± SEM (*n* = 3 per preparation)

TRL‐like particle yield of the simplified and routine procedure in terms of TG recovery was 101 ± 5% and 38 ± 12%, respectively. This difference was expected as the simplified procedure omits a selection step of a certain particle population via density gradient ultracentrifugation. The absence of this selection step resulted in higher particle heterogeneity, as reflected by a larger polydispersity index of TRL‐like particles resulting from the simplified procedure compared with that of routinely prepared particles (0.16 ± 0.003 vs 0.07 ± 0.016). Figure 1 shows the distribution of particles expressed as a percentage of the total particle number (panel a) or volume (panel b), the latter reflecting the core distribution of the content (i.e., TGs and cholesteryl esters). The mean diameter of particles prepared following the simplified procedure (57 ± 2 nm, Figure 1a; 85 ± 4 nm, Figure 1b) was smaller than that prepared using the routine procedure (74 ± 3 nm, Figure 1a; 91 ± 5 nm, Figure 1b).

### 3.2 In vivo kinetics of TRL‐like particles prepared using the simplified versus routine preparation procedure in mice at thermoneutrality

To test the *in vivo* behavior of the two preparations, we generated [^3^H]TO and [^14^C]CO double‐labeled TRL‐like particles according to both procedures. The use of these labels allowed us to simultaneously assess the tissue distribution of TG‐derived FAs (i.e., [^3^H]oleate) and the particle core (i.e., [^14^C]CO]). The plasma decay and organ uptake were first determined in mice that were housed at thermoneutrality.

**FIGURE 2:**
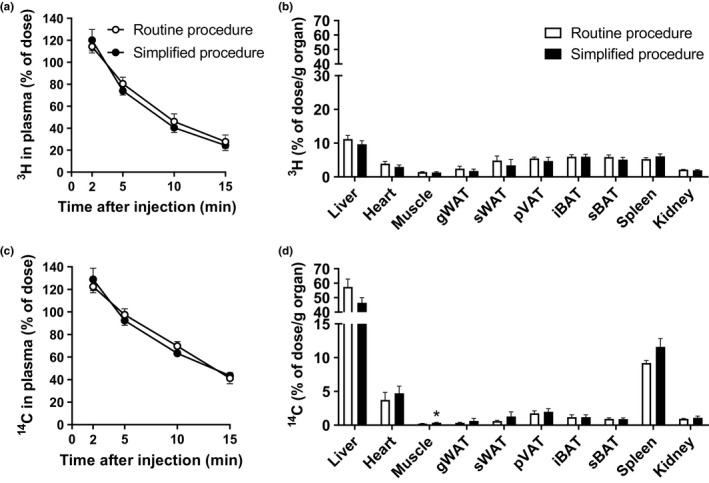
TRL‐like particles prepared following the simplified versus routine procedure show highly similar *in vivo* kinetics in mice housed at thermoneutrality. [^3^H]TO‐ and [^14^C]CO‐labeled TRL‐like particles prepared following both procedures were injected intravenously into 4 h fasted mice that were housed at thermoneutrality. Plasma at indicated time points (a, c) and organs at 15 min after injection (b, d) were assayed for ^3^H‐ and ^14^C‐activity. Values are means ± SEM (*n* = 6–8 per group). *p < 0.05 (unpaired two‐tailed Student's *t*‐test). gWAT, gonadal white adipose tissue; iBAT, interscapular BAT; pVAT, perivascular adipose tissue; sWAT, subcutaneous white adipose tissue; sBAT, subscapular BAT

Intravenous injection of the two preparations resulted in comparable plasma decay curves of both [^3^H]TO and [^14^C]CO, with [^14^C]CO decay slightly lagging (Figure 2a,c). This suggests that for both preparations, the uptake of TG‐derived FAs by organs precedes the uptake of the particle core, consistent with previous data (Khedoe et al., 2015; Rensen et al., 1997), and indicative of peripheral LPL‐mediated TG hydrolysis in metabolically active organs with the generation of remnants that are subsequently cleared by the liver. Indeed, in LPL‐expressing organs such as WAT, BAT, and skeletal muscle, [^3^H]oleate uptake exceeded the [^14^C]CO uptake by three to sixfold, whereas in the liver [^14^C]CO uptake exceeded [^3^H]oleate uptake by approx. fivefold (Figure 2b,d). The only difference between the two particle preparations was that we found a modestly higher [^14^C]CO uptake by skeletal muscle for the particles that were prepared using the simplified procedure as compared to the routine procedure (Figure 2d).

### 3.3 In vivo kinetics of TRL‐like particles prepared using the simplified versus routine preparation procedure in mice subjected to cold

**FIGURE 3:**
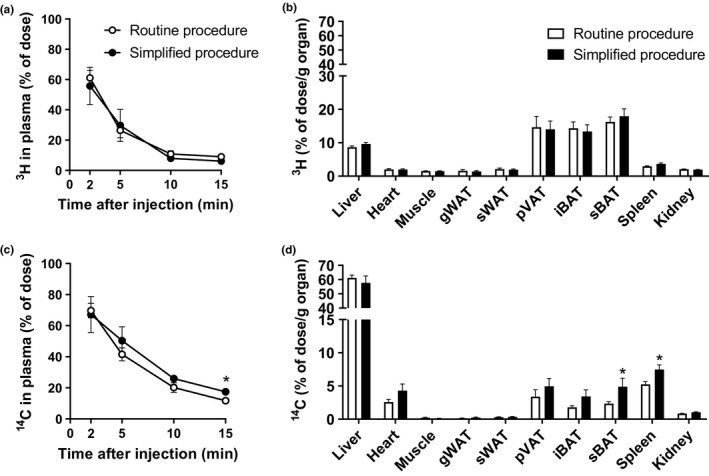
TRL‐like particles prepared following the simplified versus routine procedure show slight differences in [^14^C]CO plasma decay and organ uptake in mice subjected to cold. [^3^H]TO‐ and [^14^C]CO‐labeled TRL‐like particles prepared following both procedures were injected intravenously into 4 h fasted mice that were exposed to daily cold. Plasma at indicated time points (a, c) and organs at 15 min after injection (b, d) were assayed for ^3^H‐ and ^14^C‐activity. Values are means ± SEM (*n* = 6–8 per group). *p < 0.05 (unpaired two‐tailed Student's *t*‐test). gWAT, gonadal white adipose tissue; iBAT, interscapular BAT; pVAT, perivascular adipose tissue; sWAT, subcutaneous white adipose tissue; sBAT, subscapular BAT

Next, the *in vivo* kinetics of the preparations of the two procedures was further evaluated in mice that were subjected to a daily cold challenge, the physiological activator of BAT. Compared with thermoneutrality (Figure 2a–d), cold exposure increased the metabolic activity of BAT as reflected in higher [^3^H]oleate uptake by various BAT depots (Figure 3b) and as a consequence accelerated plasma decay of both [^3^H]TO‐ and [^14^C]CO‐derived activity (Figure 3a,c). There were no differences between TRL‐like particles that were made using the simplified or routine procedure regarding plasma decay and organ distribution of [^3^H]TO‐derived activity (Figure 3a,b). However, TRL‐like particles that were made using the simplified procedure showed a slightly delayed plasma clearance of [^14^C]CO reaching significance at 15 min (17.5% vs 11.7% of injected dose still circulating) (Figure 3c) and overall a longer plasma half‐life (6.7 min vs 4.8 min, *p* < 0.05) when compared with the routinely prepared particles. This difference was not reflected in the [^14^C]CO uptake by the liver, which was comparable for both preparations (Figure 3d). The simplified procedure also resulted in a somewhat higher [^14^C]CO uptake by the spleen and subscapular BAT, but otherwise the organ distribution was comparable (Figure 3d).

## 4 DISCUSSION

The present study shows that the simplified procedure for preparing TRL‐like particles largely shortens the preparation time, without affecting the usefulness of the particles to trace TRL metabolism *in vivo*.

While the TRL‐like particles were originally developed to selectively deliver drugs to the liver (Rensen et al., 1995), in recent years they have proven to be a useful tool to study TRL metabolism (Khedoe et al., 2015; Rensen et al., 1997), and in particular to study the effects of BAT‐modulating strategies (Berbee et al., 2015; Kooijman et al., 2015; Schilperoort et al., 2018). Since BAT was identified to be present and active in adult humans in 2007 (Nedergaard et al., 2007), activation of BAT, among other beneficial metabolic effects, has been reported to increase energy expenditure (Cypess et al., 2015), lipid turnover (Chondronikola et al., 2016), and plasma HDL‐cholesterol (Hoeke, Nahon, et al., 2017; O'Mara et al., 2020) in humans. Our group was first to show that pharmacological activation of BAT, via attenuating hypercholesterolemia, protects from atherosclerosis development in mice (Berbee et al., 2015). [^3^H]TO and [^14^C]CO double‐labeled TRL‐like particles helped us to unravel at least part of the underlying mechanism. We demonstrated that pharmacological activation of BAT increases LPL‐mediated TG‐derived FA uptake by interscapular BAT, subscapular BAT, and even perivascular adipose tissue (pVAT) that has characteristics of both WAT and BAT; these effects are accompanied by enhanced hepatic TRL‐core remnant clearance, thereby attenuating dyslipidemia, and thus atherosclerosis development (Berbee et al., 2015). More recently, using [^3^H]dipalmitoylphosphatidylcholine‐labeled TRL‐like particles, we identified that activation of BAT, through LPL‐mediated lipolysis, increases surface phospholipid transfer from TRLs to HDL (E. Zhou, Z. Li, H. Nakashima, C. Liu, Z. Ying, A. C. Foks, J. F. P. Berbée, K. W. van Dijk, P. C. N. Rensen, & Y. Wang, unpublished data), accompanied by enhanced HDL functionality, and reverse cholesterol transport (Bartelt et al., 2017). Of interest, using [^3^H]TO‐labeled TRL‐like particles, we also found that BAT exhibits a pronounced circadian rhythm in TG‐derived FA uptake that was reflected in plasma TG concentrations (Berg et al., 2018), which suggests that time of day might play a role in the effectiveness of BAT‐activating strategies aimed to lower plasma lipid concentrations.

TRL‐like particles that were prepared following the simplified procedure showed similar plasma clearance of [^3^H]TO and uptake of [^3^H]TO‐derived [^3^H]oleate by BAT and other metabolically active tissues when compared to particles prepared by the routine procedure. Exposing mice to cold accelerated plasma clearance of [^3^H]TO due to enhanced uptake of [^3^H]oleate by BAT in a similar way for both preparations. These observations are important as they open up the way for the use of TRL‐like particles to study BAT activity in humans. Currently, uptake of [^18^F]fluorodeoxyglucose visualized by PET‐CT is the most widely applied technique to determine BAT volume and activity in humans (Cohade et al., 2003; Cypess et al., 2009, 2015). However, we have shown that murine BAT exhibits preferred uptake of TG‐derived FAs over glucose (Li et al., 2018; Schilperoort et al., 2018) and that cold exposure—the physiological activator of BAT—enhances lipid oxidation rather than glucose oxidation in humans (Bakker et al., 2014). These data are in line with the fact that FAs rather than glucose are the main substrate for BAT thermogenesis (Ouellet et al., 2012; Schilperoort et al., 2016). In addition, the use of glucose as a tracer may cause an underestimation of BAT volume and activity in subjects with obesity and diabetes as well as in older persons, due to the development of insulin resistance that hampers [^18^F]fluorodeoxyglucose uptake by BAT (Blondin et al., 2015). Even though insulin resistance may also dampen the uptake of TG‐derived FAs because of decreased LPL activity (Qu et al., 2016), we do expect that determining the uptake of TG‐derived FAs as a measure of BAT volume and activity will greatly enhance the sensitivity and specificity. Therefore, we anticipate that PET‐compatible TG tracers that are incorporated in TRL‐like particles will provide a valuable tool to measure BAT volume and activity in humans. The first step toward the use of PET‐compatible TG tracers was to simplify and shorten the preparation procedure of TRL‐like particles as the half‐lives of PET tracers are typically short (e.g., 110 min for ^18^F). Using the simplified procedure as described within this study, the preparation time of TRL‐like particles was reduced from over 4 h to less than 2 h. By first evaporating solvents of the non‐radiolabeled lipids before adding radiolabeled lipid‐based tracers, the time from preparation to injection may even be further reduced.

Generation of a PET‐compatible TG tracer will also be advantageous over the FA tracer [^18^F]fluoro‐6‐thia‐heptadecanoic acid (FTHA). Although [^18^F]FTHA has been used to trace BAT (Blondin et al., 2015; Ouellet et al., 2012), the uptake by BAT is low since FAs taken up by BAT are mainly derived from TRL‐derived TG (Festuccia et al., 2011) and the majority binds to albumin and is scavenged by the liver (Ouellet et al., 2012).

While no differences were found in the [^3^H]TO plasma clearance and [^3^H]oleate uptake by organs, the plasma clearance of [^14^C]CO incorporated in TRL‐like particles prepared using the simplified procedure was slower in mice exposed to cold than that of routinely prepared particles. This is probably due to the fact that the average size of the TRL‐like particles generated by the simplified procedure was smaller. Indeed, by comparing *in vivo* kinetics of [^14^C]CO incorporated in TRL‐like particles of different size (i.e., a mean diameter of 45, 75, and 150 nm, respectively), we previously reported that smaller particles are characterized by a slower plasma clearance (Khedoe et al., 2015; Rensen et al., 1997). In addition, the newly prepared particles showed a higher [^14^C]CO uptake by the spleen, which is likely due to the presence of also large particles in the more heterogeneous emulsion (Khedoe et al., 2015; Rensen et al., 1997). We expect that the minor differences caused by the smaller average particle size and increased heterogeneity of the emulsion particles prepared according to the simplified procedure will have a minimal effect when using them to study TLR metabolism.

To shorten preparation time, we omitted fractionation of TRL‐like particles using density gradient ultracentrifugation from the preparation procedure. This may limit their use in the discrimination between LDLr‐dependent and LDLr‐independent pathways involved in TRL‐remnant uptake. Using LDLr^−/−^ and wild‐type mice, Rensen et al. (1997) previously identified that liver uptake of 50 nm TRL‐like particles is almost exclusively mediated by LDLr, while 150 nm particles are mainly taken up via other hepatic receptors. Therefore, by comparing liver uptake of 50 and 150 nm TRL‐like particles, contribution of LDLr‐dependent and LDLr‐independent pathways to liver TRL‐remnant clearance can be studied. Unlike the routine procedure which produces three fractions of TRL‐like particles with mean diameters of 150, 75, and 50 nm via subsequent steps of density gradient ultracentrifugation (Rensen et al., 1997), the simplified procedure only yields a single particle emulsion, and cannot be used to study the contribution of the LDLr to hepatic TRL‐remnant clearance, for example, in context of a PCSK9 inhibitor that specifically increases hepatic LDLr levels (Hoeke, Wang, et al., 2017).

In conclusion, we present a simplified procedure that shortens the preparation time of TRL‐like particles for tracing TRL metabolism *in vivo*. Time saving associated with the new procedure will allow for incorporating short‐lived PET‐compatible TG tracers into TRL‐like particles, which may prove to be superior tracers compared to the currently widely used [^18^F]fluorodeoxyglucose tracer for assessing human BAT volume and activity in the near future.

## CONFLICT OF INTEREST

This work was also supported by a Lilly Research Award Program (LRAP) Award (to P.C.N.R.). TC is an employee and shareholder of Eli Lilly and Company. Eli Lilly and Company had no role in study design, data collection and analysis, decision to publish, or preparation of the manuscript.

## AUTHOR CONTRIBUTIONS

Zhixiong Ying: Conceptualization; Investigation, Data acquisition and analysis; Writing of the original draft and review. Mariëtte R. Boon: Conceptualization, Investigation, Funding acquisition. Tamer Coskun: Resources. Sander Kooijman: Investigation, Supervision, Writing and editing. Patrick C.N. Rensen: Conceptualization, Project administration, Supervision, Writing and editing.

## Data Availability

The datasets used and/or analyzed during the current study are available from the corresponding author on reasonable request.
